# A New Medical College Proposed

**Published:** 1890-10

**Authors:** Thomas A. Pope

**Affiliations:** Cameron, Texas


DANIEL'S

### TEXAS MEDICAL JOURNAL.

A Representative Organ of the Medical Profession, and an Exponent of Rational Medicine; devoted to the Organization, Advancement and Elevation of the Profession in Texas.

PUBLISHED MONTHLY.—SUBSCRIPTION $2.00 A YEAR.

Vol. VI. AUSTIN, OCTOBER, 1890. No. 4.

### Original Articles.

CONTRIBUTED EXCLUSIVELY TO THIS JOURNAL.

*The Articles in this Department are accepted and published with the understanding that we are not responsible for, nor do we indorse the views and opinions of the writers by so doing.*

---

### A NEW MEDICAL COLLEGE PROPOSED—IS IT WISE?

BY THOMAS A. POPE, M. D., CAMERON, TEXAS.

[In transmitting the following communication, Dr. Pope says: "Enclosed is a resumé of a bill which will be published shortly. If you choose you can publish it over my signature. I know that no man in Texas has worked more faithfully than you to elevate the professional standard; and you are in a position to help the good cause as effectually as any man in Texas, if not more so." Following is the communication—ED: ]

*Editor Daniel's Texas Medical Journal:*

BEFORE the query can be intelligently answered its antecedents must be known and understood. Then what of the new College of Medicine? Briefly this: A bill is proposed which, if it become a law, would create a State Board of Health and establish and charter the "College of Physicians and Surgeons of Texas," and locate the same at Austin.

The College would not be a teaching school, or give any course of lectures. The Faculty would be composed of ten Professors, one for each of the seven branches of medicine, one of Hygiene and State Medicine, one of Homeopathy, and one of the Eclectic system of medicine. The State quarantine officer would be *ex-officio* member of the Faculty, Secretary of the State Board of Health and College, and Professor of Hygiene and State Medicine. The first Faculty would be nominated by the Texas State Medical Association, and should serve five years. All vacancies in the Faculty to be filled by the Fellows of the College. The Faculty of the College constitutes a State Board of Health, that meets annually, and looks after quarantine matters, epidemics, sanitary science, and adulterations of food and medicine.

The law will provide that all births and deaths shall be recorded by County Clerks. The cause of death, age, color and nativity of deceased shall also be recorded, and the Clerks are required to transmit a transcript of such records annually to the State Board of Health for publication.

There shall be at least one session annually of the College, to be held in Austin, beginning on the first Monday in April. The College is authorized to confer the degree of Doctor of Medicine. Every candidate for a degree must be examined in all the seven branches of medicine and sanitary science; provided, that applicants belonging to Homeopathic or Eclectic schools shall be examined by the Professors of those schools in Materia Medica and Therapeutics, and Theory and Practice of Medicine. Those passing examination and receiving degrees shall be Fellows of the College, and eligible for appointment as Professors in said College.

After the passage of the act it shall not be lawful for any one to *begin* and pursue the practice of medicine without first having obtained the degree of Doctor of Medicine from the College.

Two years after the passage of the act no one except Fellows of the College will be permitted to testify as experts in any court in the State, or be employed by State, county or municipality as a physician. And further, two years after the passage of the act, no charge for medical services can be collected by law unless the party rendering the service is a Fellow of the College, or unless the party to be charged has promised in writing to pay the same.

The College can issue license authorizing the holder to practice midwifery.

Many other provisions have not been mentioned, because I do not wish to make this article so long that it will not be read. The salient points given above will acquaint the reader somewhat with the bill, and perhaps induce him to obtain a copy and examine it carefully.

Now, we again ask the question, is it wise? That the State should have a Board of Health to gather statistics and protect the health of our citizens, every one must agree, and so we conclude that part of the proposed law would be heartily endorsed by the medical profession and the people. Then as to the College. The time is coming when the State will be bound to take some action to protect its citizens from the quackery of some, and the ignorance and stupidity of others, who are now, or will be practicing medicine in Texas, and it seems to me that the step might well be taken now. Some efforts have already been made in this direction, but have proved to be lamentable failures, although some good was no doubt accomplished.

This law will kill quackery absolutely and forever in Texas. That is, the traveling kind. It will shut out every incompetent person who might in the future wish to locate in Texas to practice medicine. It will raise the professional standard so high in Texas that to be a physician in good standing here will be an honor that the whole world will be quick to recognize and appreciate. The only effect it will have on those now practicing in Texas will be so make better physicians of them all. My hair is turning white, and I have practiced medicine for many years, and yet if such a college is established I shall hunt up my text books and spend a solid year, or two or three, if necessary, in going over again all the ground-work and bringing myself fully up with the medical knowledge of to-day, and then I would go to Austin and say, "I am ready. Examine me, and if I am competent, grant me the honor of a degree." And so I believe it would be with every physician in Texas who loves his profession and does not wish to lag behind, and out of the five thousand doctors in Texas, if you could devise some way to put four thousand of them to studying hard for a couple of years, more medical knowledge would be absorbed in Texas within that time than was ever acquired in any ten years before, and the gain to the profession would be immense, and to the patients immensely greater.

The law, as stated, would not interfere with those now practicing in Texas, except that they would not be allowed to testify as experts, or be employed by the State as physicians, and yet with proper care a diploma from the contemplated College will confer the highest honor, and be a guarantee that the holder is entitled to rank with the best in any land, in medical knowledge. In England the college from which a degree is prized more highly than any other college among English-speaking people, is not a teaching school. No lectures are given by the Faculty, and any one who aspires may apply for a degree of Doctor of Medicine, which the State authorizes it to grant.

It is probable that an inter-leaved copy of the proposed law will be printed, and any physician who will send me his address can have a copy, and he can place his criticisms and suggestions along side of the original, and thus the opinions of all may be consulted and their suggestions utilized, until a perfect bill can be fashioned. The only aim of the author is to elevate the profession and promote the welfare of the people, and to this object every unselfish, intelligent man in Texas, whether a physician or not, will certainly give a cordial support. It is only a question of method. Whenever we can become united on some fair, just and reasonable basis, the battle is won.

